# An impaired pituitary–adrenal signalling axis in stable cirrhosis is linked to worse prognosis

**DOI:** 10.1016/j.jhepr.2023.100789

**Published:** 2023-05-11

**Authors:** Lukas Hartl, Benedikt Simbrunner, Mathias Jachs, Peter Wolf, David Josef Maria Bauer, Bernhard Scheiner, Lorenz Balcar, Georg Semmler, Michael Schwarz, Rodrig Marculescu, Michael Trauner, Mattias Mandorfer, Thomas Reiberger

**Affiliations:** 1 Division of Gastroenterology and Hepatology, Department of Medicine III, Medical University of Vienna, Vienna, Austria; 2 Vienna Hepatic Hemodynamic Lab, Division of Gastroenterology and Hepatology, Department of Medicine III, Medical University of Vienna, Vienna, Austria; 3 Christian Doppler Lab for Portal Hypertension and Liver Fibrosis, Division of Gastroenterology and Hepatology, Department of Medicine III, Medical University of Vienna, Vienna, Austria; 4 Division of Endocrinology, Department of Medicine III, Medical University of Vienna, Vienna, Austria; 5 Department of Laboratory Medicine, Medical University of Vienna, Vienna, Austria

**Keywords:** Cortisol, ACTH, Bile acids, Inflammation, Advanced chronic liver disease, Portal hypertension, Non-invasive testing, Cirrhosis

## Abstract

**Background & Aims:**

Inadequate adrenal function has been described in patients with cirrhosis. We investigated (i) the pituitary–adrenal axis at different clinical stages and (ii) the clinical impact of decreased serum cortisol levels in stable patients with advanced chronic liver disease (ACLD).

**Methods:**

We included 137 outpatients with ACLD undergoing hepatic venous pressure gradient (HVPG) measurement in the prospective VICIS study (NCT03267615). Patients were stratified into six clinical stages: S0: subclinical portal hypertension (PH) (HVPG 6–9 mmHg), S1: clinically significant PH (HVPG ≥10 mmHg) without varices, S2: presence of varices, S3: previous variceal bleeding, S4: previous non-bleeding decompensation, and S5: further decompensation.

**Results:**

Fifty-one patients had compensated ACLD (S0: n = 13; S1: n = 12; S2: n = 26), whereas 86 patients had decompensated ACLD (S3: n = 7; S4: n = 46; S5: n = 33). Serum total cortisol (t-Cort) showed a strong correlation with estimated serum free cortisol (f-Cort; Spearman’s ρ: 0.889). With progressive clinical stage, median ACTH levels (from S0: 44.0 pg/ml to S5: 20.0 pg/ml; *p* = 0.006), t-Cort (from S0: 13.9 μg/dl to S5: 9.2 μg/dl; *p* = 0.091), and cortisol binding globulin (from S0: 49.3 μg/ml to S5: 38.9 μg/ml; *p* <0.001) decreased, whereas f-Cort (*p* = 0.474) remained unchanged. Lower t-Cort levels independently predicted bacterial infections (asHR: 1.11; 95% CI: 1.04–1.19; *p* = 0.002), further decompensation (asHR: 1.08; 95% CI: 1.02–1.12; *p* = 0.008), acute-on-chronic liver failure (ACLF; asHR: 1.11; 95% CI: 1.04–1.19; *p* = 0.002), and liver-related death (asHR: 1.09; 95% CI: 1.01-1.18; *p* = 0.045).

**Conclusions:**

The pituitary–ACTH–adrenal–cortisol axis is progressively suppressed with increasing severity of cirrhosis. Lower t-Cort is an independent risk factor for bacterial infections, further decompensation of ACLF, and liver-related mortality—even in stable outpatients with cirrhosis.

**Clinical trial number:**

Vienna Cirrhosis Study (VICIS; NCT: NCT03267615).

**Impact and Implications:**

In a cohort of stable outpatients, we observed progressive suppression of the pituitary–adrenal axis with increasing clinical stage of advanced chronic liver disease (ACLD). Increased levels of bile acids and systemic inflammation (assessed by interleukin-6 levels) could be involved in this suppression. Serum total cortisol (t-Cort) was strongly correlated with serum free cortisol (f-Cort) and lower t-Cort levels were independently associated with a higher risk of adverse clinical outcomes, including bacterial infections, further decompensation, acute-on-chronic liver failure, and liver-related death.

## Highlights

• Adrenocorticotropic releasing hormone (ACTH) declines throughout the substages of advanced chronic liver disease (ACLD).
• Plasma total cortisol (t-Cort) decreases with severity of ACLD.
• Low estimated serum free cortisol (f-Cort; <4.8 ng/ml) increases with advancing ACLD.
• t-Cort and f-Cort correlate strongly in stable outpatients with ACLD.
• Lower t-Cort is a risk factor for acute-on-chronic liver failure and liver-related death.

## Graphical abstract

**Figure:**
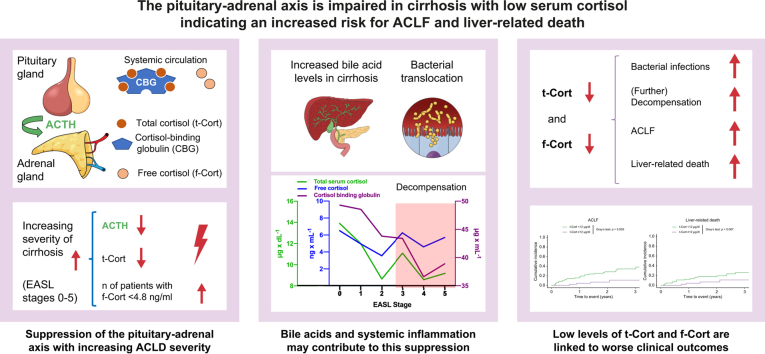

## Introduction

Advanced chronic liver disease (ACLD) causes considerable morbidity and mortality on a global scale.^1^^,^^2^ In the early stages, that is in the absence of complications, ACLD is considered compensated (cACLD). Eventually, patients can develop complications related to portal hypertension, such as ascites, variceal bleeding, or hepatic encephalopathy, which are indicators of decompensated ACLD (dACLD).[3], [4], [5] The first decompensation marks an important event, as patients with dACLD have a significantly worse prognosis than patients with cACLD.^6^^,^^7^

Moreover, acute-on-chronic liver failure (ACLF) has been established as a severe complication of ACLD^6^^,^^8^ characterised by extrahepatic organ failure(s), and is associated with dismal clinical outcomes.^9^^,^^10^ ACLF is closely linked to systemic inflammation^11^ and is frequently triggered by precipitating events, mostly alcoholic hepatitis and bacterial infections.^12^

Generally, patients with ACLD are at increased risk of serious infections.^13^ Although different factors are involved, including bacterial translocation,^14^ intestinal dysbiosis and the development of cirrhosis-associated immune deficiency,^15^ dysfunction of the pituitary–adrenal axis may represent another predisposing factor.

Altered cortisol metabolism in cirrhosis has already been described in 1967,^16^ linking chronic liver disease to adrenocortical dysfunction. One recent study investigated adrenal function in patients with ACLD hospitalised for acute decompensation, finding that relative adrenal insufficiency was frequent in these patients and had similar impact on the prognosis as did other organ failures as defined by ACLF.^17^ Accordingly, another study indicated decreased adrenal function by exploring decreased maximal cortisol secretion rates in patients with ACLD and particularly in patients with Child–Turcotte–Pugh (CTP) stages B and C.^18^ A small study indicated that this adrenal insufficiency may be a result of dysregulated pituitary signalling, as direct adrenal stimulation with adrenocorticotropic releasing hormone (ACTH) yielded significantly better results than indirect stimulation.^19^^,^^20^ Mechanistically, elevated bile acid levels^21^^,^^22^ and chronic inflammation^23^ may impair the hypothalamus–pituitary–adrenal signalling axis.

Nonetheless, abnormalities of the pituitary–adrenal axis have not been systematically investigated in the different substages of cirrhosis. Thus, this study aimed (i) to investigate the pituitary–adrenal axis throughout individual clinical stages of ACLD^6^ and (ii) to determine the impact of serum total cortisol (t-Cort) and estimated serum free cortisol (f-Cort) levels on clinical outcomes of ACLD.

## Patients and methods

### Study design

This prospective study investigated consecutive patients with ACLD and portal hypertension (*i.e.* HVPG ≥6 mmHg) who underwent HVPG measurement at the Vienna Hepatic Hemodynamic Laboratory (Division of Gastroenterology and Hepatology, Medical University of Vienna) between June 2018 and March 2020 under standardised conditions.^24^ We only included clinically stable outpatients in whom data on t-Cort and ACTH were available.

Patients with occlusive portal vein thrombosis (PVT), a history of vascular liver disease, hepatocellular carcinoma (HCC), liver transplantation (LT), transjugular intrahepatic portosystemic shunt (TIPS), human immunodeficiency virus, or active infection at the time of HVPG measurement were excluded. Additional exclusion criteria included: therapy with corticosteroids or other medications that influence steroidogenesis, ongoing significant^25^ self-reported alcohol intake at characterisation, and HVPG measurement while on non-selective beta blocker treatment.

HVPG measurements and central venous blood withdrawals were conducted under fasting conditions to evaluate cortisol axis parameters. Information was obtained on the development of clinically apparent ascites, variceal bleeding, hepatic encephalopathy (HE), ACLF, acute kidney injury (AKI), and HCC, as well as LT and (liver-related) death during clinical follow-up (FU).

### Patient cohorts

For the baseline characterisation of the pituitary–adrenal axis in patients with ACLD, patients were stratified according to ACLD severity and clinical stages as proposed by D’Amico *et al.* in 2018.^5^^,^^6^ The absence of any previous decompensating event defined cACLD, which was further subdivided into three distinct stages (S): S0: subclinical portal hypertension (PH; HVPG 6–9 mmHg), S1: clinically significant PH (*i.e.* HVPG ≥10 mmHg) without the presence of varices, and S2: presence of varices. In contrast, patients with a history of at least one decompensating event were classified as patients with dACLD with three substages: S3: history of decompensation from acute variceal bleeding, and S4: non-bleeding decompensation events including ascites and HE, as well as S5: further decompensation defined as history of two different types of decompensation events.

### Decompensation events

For patients with cACLD, decompensation was defined as the occurrence of moderate or large ascites, variceal bleeding, or clinically overt hepatic encephalopathy. In patients with dACLD, further decompensation was defined as the occurrence of a second type of hepatic decompensation or worsening of the first hepatic decompensation. Worsening included development of refractory ascites or spontaneous bacterial peritonitis in patients with ascites, first hospital admission with HE in patients with H3, and variceal rebleeding in patients with a history of variceal haemorrhage. Finally, ACLF as defined by the EASL-Chronic Liver Failure (EASL-CLIF) consortium definition^8^^,^^26^ was regarded as a fourth and distinct type of decompensation event, which could potentially occur in both cACLD, and dACLD patients.

### Assessment of HVPG and liver stiffness measurement

HVPG measurements were conducted according to a standardised operating procedure, as previously described.^24^ Briefly, after local anaesthesia, a catheter introducer sheath was positioned in the right internal jugular vein. Subsequently, a specifically designed balloon catheter^27^ was inserted under fluoroscopic guidance into a large hepatic vein. HVPG was calculated by subtracting the free pressure from the wedged hepatic vein pressure. A total of three measurements were performed per patient and the mean HVPG of these measurements was used for further analyses.

As previously described, liver stiffness measurement (LSM) was assessed by vibration-controlled transient elastography (FibroScan®; Echosens, Paris, France)^28^ under fasting conditions.

### Cortisol axis parameters and routine laboratory parameters

Laboratory tests were conducted in the ISO-certified Department of Laboratory Medicine of the Medical University of Vienna. t-Cort (normal range for morning t-Cort: 6.2–18.0 μg/dl) was analysed by electrochemiluminescence immunoassay (Elecsys Cortisol II, Roche Diagnostics GmbH, Mannheim, Germany), with precision comparable to gold standard liquid chromatography–tandem mass spectrometry.^29^ Cortisol binding globulin (CBG; normal range: 22.0–55.0 μg/ml for men and 40.0–154.0 μg/ml for women) was measured by radio immunoassay (CBG-RIA-CT, DIAsource immunoassays, Nivelles, Belgium), ACTH (normal range: 7.2–63.3 pg/ml; Elecsys ACTH, Roche Diagnostics GmbH, Mannheim, Germany), as well as aldosterone (normal range: 12.0–236.0 pg/ml; Liaison XL, DiaSorin, Saluggia, Italy) were determined by chemiluminescence immunoassays and corticosterone (normal range: 2.3–10.8 ng/ml) by enzyme immunoassay (Corticosterone ELISA, DRG International Inc., Springfield, NJ, USA). f-Cort (normal range for morning f-Cort: 4.3–17.7 ng/ml) was calculated from t-Cort and CBG levels as previously described.^30^ Bile acids (normal range: 0.0–10.0 μmol/L; consisting of unconjugated acids, taurine- and glycine-conjugated acids) were measured by high-performance liquid chromatography–high-resolution mass spectrometry (HPLC–HRMS; Q Exactive MS/MS, ThermoFisher Scientific, Waltham, MA, USA) as previously described.^31^^,^^32^ Standard laboratory methods were used for the assessment of routine laboratory parameters.

Blood withdrawals were conducted in a standardised manner after HVPG measurement under fasting conditions and after resting in the supine position for at least 30 min. All blood samples for assessment of the parameters of the pituitary–adrenal axis were taken between 9 am and 12 noon.

### Statistical analysis

For categorical variables, the number (n) and proportion (%) of patients displaying the parameter of interest were reported. The median and IQR were used to present continuous data. The Mann–Whitney *U* test was implemented for comparison of continuous non-normally distributed variables between two different groups. The Kruskal–Wallis test was performed for comparison of continuous variables in three of more groups. Pearson’s Chi squared test and Fisher’s exact test were used for group comparisons of categorical variables, as appropriate. Correlations were assessed using Spearman’s Rho (ρ).

Clinical outcomes at the 3-year follow-up were assessed by cumulative incidence calculation. Cumulative incidence comparison was performed using Gray’s test, as previously described.^33^ Fine and Gray competing risk regression models were calculated using the R package cmprsk^33^^,^^34^ (R Foundation for Statistical Computing, Vienna, Austria) to evaluate whether t-Cort and f-Cort levels were associated with the risk of clinical events of interest. Apart from t-Cort or f-Cort, well-established risk factors for worse outcomes in ACLD (*i.e.* age, CTP score, serum creatinine, sodium, HVPG as a marker for portal hypertension, and C-reactive protein (CRP) as a parameter of systemic inflammation) and sex were evaluated by univariate and multivariate analysis. Parameters that conferred prognostic value in univariate regression analysis (as evidenced by *p* <0.100) were carried forward to multivariate regression analysis. The exception was f-Cort as a key parameter of interest, which was carried forward to test its potential stage-specific prognostic information after adjustment for relevant parameters (particularly liver function, *i.e.* CTP score). LT and non-liver-related death were considered as competing risks for decompensation/further decompensation and liver-related death, whereas LT and death were considered competing events for bacterial infections and ACLF for both the cumulative incidence comparison and the competing risk regression. Youden’s index was used to determine the optimal cut-off values for t-Cort and f-Cort for the prediction of ACLF, as a close link between this critical condition and adrenal function has been previously reported.^17^

Statistical analyses were performed using GraphPad Prism v8 (GraphPad Software, La Jolla, CA, USA), IBM SPSS v27.0 statistic software (IBM, Armonk, NY, USA) and R v4.2.1 (R Core Team). A two-sided value of *p* <0.05 was considered statistically significant.

### Ethics

The study was approved by the Ethics Committee of the Medical University of Vienna (No. 1262/2017). It was performed in accordance with the current version of the Declaration of Helsinki. All included patients are part of the Vienna Cirrhosis Study (VICIS; NCT: NCT03267615) and gave their written informed consent before inclusion in the study.

## Results

### Patient characteristics

**Fig. 1:**
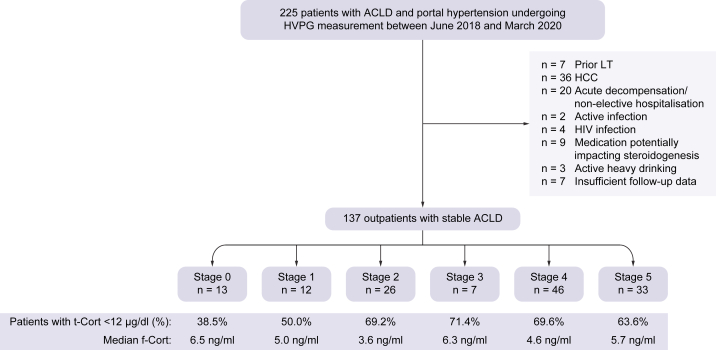
Patient flow-chart. ACLD, advanced chronic liver disease; f-Cort, serum free cortisol; HCC, hepatocellular carcinoma; HVPG, hepatic venous pressure gradient; LT, liver transplantation; t-Cort, serum total cortisol.

In total, 137 patients with ACLD (67.9% males) and a median age of 58.1 years were included in the study. Fig. 1 depicts the cohort building process, and Table 1 shows the baseline characteristics of the included patients. The main aetiology was alcohol-related liver disease (47.4%), followed by viral hepatitis (14.6%), and non-alcoholic steatohepatitis (NASH; 12.4%). Among patients with chronic alcohol consumption (ALD and mixed aetiologies), 84.4% (n = 65/77) reported being abstinent at the time of characterisation, whereas 12 patients reported continuing moderate alcohol consumption. In total, 62.8% of the included patients had dACLD, which was most frequently associated with ascites (56.2%). The median model for end-stage liver disease (MELD) was 12.0 points and the median HVPG was 16 mmHg.

**Table 1:** Patient characteristics.

Patient characteristics	All patients (N = 137)
Sex, male/female (% male)	93/44 (67.9%)
Age, years (IQR)	58.1 (50.0–65.6)
BMI, kg/m^2^ (IQR)	26.0 (23.5–29.6)
Aetiology	
ALD, n (%)	65 (47.4%)
Viral hepatitis, n (%)	20 (14.6%)
ALD + viral hepatitis, n (%)	12 (8.8%)
NASH, n (%)	17 (12.4%)
Cholestatic, n (%)	1 (0.7%)
Other, n (%)	22 (16.1%)
Decompensated ACLD, n (%)	86 (62.8%)
Ascites, n (%)	77 (56.2%)
EASL stage	
0, n (%)	13 (9.5%)
1, n (%)	12 (8.8%)
2, n (%)	26 (19.0%)
3, n (%)	7 (5.1%)
4, n (%)	46 (33.6%)
5, n (%)	33 (24.1%)
MELD, points (IQR)	12.0 (9.0-15.0)
CTP score, points (IQR)	6.0 (5.0-8.0)
CTP stage	
A, n (%)	71 (51.8%)
B, n (%)	52 (38.0%)
C, n (%)	14 (10.2%)
Bilirubin, mg/dl (IQR)	1.1 (0.7–1.9)
Albumin, g/dl (IQR)	37.0 (33.5–40.3)
INR, units (IQR)	1.4 (1.2–1.5)
Sodium, mmol/L (IQR)	139.0 (136.5–140.0)
HVPG, mmHg (IQR)	16 (11–20)
MAP, mmHg (IQR)	101 (91–110)
LSM, kPa (IQR)	35.1 (20.2–57.1)
Serum total cortisol, μg/dl (IQR)	10.0 (6.2–13.2)
Cortisol binding globulin, μg/ml (IQR)^1^	41.5 (34.4–47.5)
Serum free cortisol, ng/ml (IQR)^1^	5.0 (2.9–7.2)
Bile acids, μmol/L (IQR)	14.2 (6.5–41.4)
Total cholesterol, mg/dl (IQR)	139.0 (117.5–167.5)
HDL cholesterol, mg/dl (IQR)	44.0 (35.5–55.0)
LDL cholesterol, mg/dl (IQR)	77.2 (58.5–100.8)
Statin intake, n (%)	21 (9.9%)
IL-6, ng/dl (IQR)	8.0 (4.0–15.5)
WBC, G/L (IQR)	4.9 (3.6–6.2)
CRP, mg/dl (IQR)	0.3 (0.1–0.8)

ACLD, advanced chronic liver disease; ALD, alcohol-related liver disease; CTP, Child-Turcotte-Pugh; CRP, C-reactive protein; HVPG, hepatic venous pressure gradient; IL-6, interleukin-6; INR, international normalized ratio; LSM, liver stiffness measurement; LT, liver transplantation; MAP, mean arterial pressure; MELD, model for end-stage liver disease; NASH, non-alcoholic liver disease; WBC, white blood count.

1 available in 133 patients.

### Pituitary–adrenal–axis in patients with different clinical stages of ACLD

**Fig. 2:**
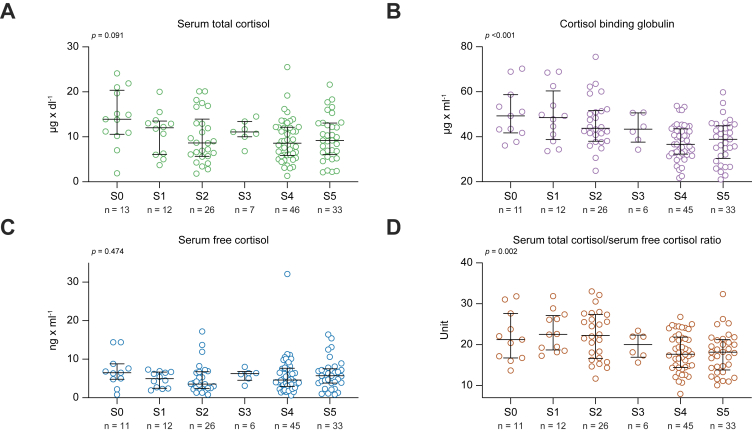
**Plasma levels of serum total and free cortisol and cortisol binding globulin**. Plasma levels of (A) serum total cortisol, (B) cortisol binding globulin, (C) serum free cortisol and (D) serum total cortisol/serum free cortisol ratio in patients with different stages of ACLD. Patients stratified by EASL stages. Levels of significance of group comparisons determined by the Kruskal–Wallis test: (A) *p* = 0.091, (B) *p* <0.001, (C) *p* = 0.474, and (D) *p* = 0.002. The median and interquartile range for every group is shown.

**Fig. 3:**
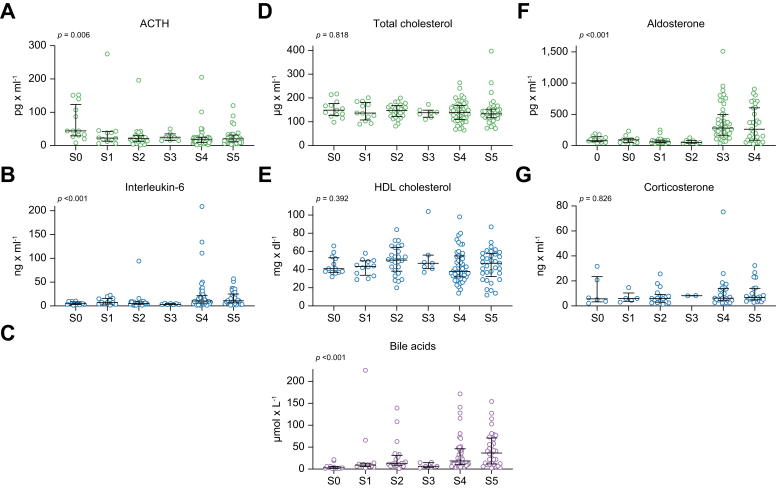
**Plasma levels of components of the pituitary-adrenal axis, systemic inflammation and bile acids**. Plasma levels of (A) adrenocorticotropic releasing hormone (ACTH), (B) interleukin-6, (C) bile acids, (D) total cholesterol, (E) HDL cholesterol, (F) aldosterone and (G) corticosterone in patients with different stages of ACLD. Patients stratified by EASL stages. Levels of significance of grou*p* comparisons by Kruskal-Wallis test: (A) *p* = 0.006, (B) *p* <0.001, (C) *p* <0.001, (D) *p* = 0.818, (E) *p* = 0.392, (F) *p* <0.001, and (G) *p* = 0.826. The median and interquartile range for every grou*p* is provided.

With progressive severity of ACLD, patients had lower levels of t-Cort (from S0: 13.9 μg/dl to S5: 9.2 μg/dl; *p* = 0.091) (Fig. 2; Table S1). However, there were also lower median levels of CBG with increasing clinical stage of ACLD (from S0: 49.3 μg/ml to S5: 38.9 μg/ml; *p* <0.001), resulting in overall unchanged levels of f-Cort throughout the ACLD substages (*p* = 0.474). The t-Cort/f-Cort ratio decreased significantly with the clinical stage of ACLD, particularly in patients with dACLD (from S0: 21.2 to S5: 18.2; *p* = 0.002). Furthermore, ACTH decreased markedly across the different ACLD substages (from S0: 44.0 pg/ml to S5: 20.0 pg/ml; *p* = 0.006) (Fig. 3). Furthermore, corticosterone levels did not differ across the clinical stages (from S0: 6.0 ng/ml to S5: 6.7 ng/ml; *p* = 0.826), whereas aldosterone levels increased, particularly in patients with ACLD with non-bleeding decompensation (from S0: 78.0 pg/ml to S4: 280.0 pg/ml and to S5: 262.0 pg/ml; *p* <0.001). Table S2 shows the levels of parameters of the pituitary–adrenal axis in cACLD compared with dACLD.

In particular, total cholesterol (from S0: 149.0 mg/dl to S5: 133.0 mg/dl; *p* = 0.818) did not decrease significantly with the progression of the clinical stage of ACLD and HDL (from S0: 41.0 mg/dl to S5: 47.0 mg/dl; *p* = 0.392) remained mostly unchanged. Bile acids (from S0: 2.7 μmol/L to S5: 36.5 μmol/L; *p* <0.001) and interleukin-6 (IL-6; from S0: 4.7 ng/dl to S5: 10.8 ng/dl; *p* <0.001) increased significantly with ACLD severity.

### Correlations of f-Cort and other parameters of the pituitary–adrenal axis

**Fig. 4:**
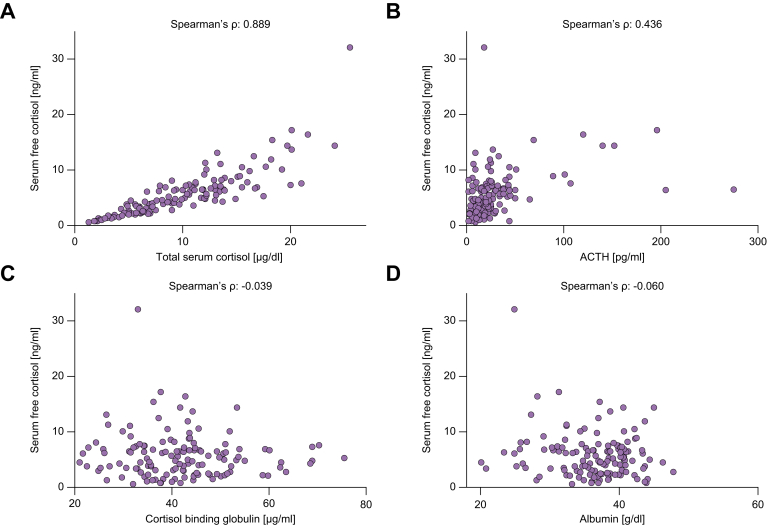
**Correlations between serum free cortisol and other parameters of the pituitary-adrenal axis**. Correlations of serum free cortisol and (A) serum total cortisol, (B) adrenocorticotropic releasing hormone (ACTH), (C) cortisol binding globulin and (D) albumin. Correlation coefficients as assessed by Spearman’s ρ with 95% confidence intervals (95% CI) and levels of significance: (A) ρ = 0.889 (95% CI: 0.846–0.921), *p* <0.001; (B) ρ=0.436 (95% CI: 0.282–0.567), *p* <0.001; (C) ρ = -0.039 (95% CI: -0.212 to 0.137), *p* = 0.659; (D) ρ = -0.060 (95% CI: -0.233 to 0.116), *p* = 0.489.

t-Cort and f-Cort levels showed a strong correlation in our cohort of stable outpatients with ACLD and PH (Spearman’s ρ: 0.889; *p* <0.001) (Fig. 4). At the same time, f-Cort did not correlate with CBG (Spearman’s ρ: -0.039; *p* = 0.659) or albumin (Spearman’s ρ: -0.060; *p* = 0.489). Finally, there was a moderate correlation between f-Cort and ACTH (Spearman’s ρ: 0.436; *p* <0.001).

### Follow-up and clinical outcomes

The median FU time was 661.0 days. Overall, 41.6% of the patients (n = 57) experienced at least one further decompensation event. Table 2 details the prevalence of different decompensation events. Three patients (2.2%) developed HCC during follow-up. TIPS implantation was performed in 8.0% (n = 11) of the patients and 11 patients (8.0%) underwent liver transplantation. Approximately 23.4% (n = 32) of the patients died, and 24 of these deaths (75.0%) were liver related.

**Table 2:** Clinical outcomes.

Clinical outcomes	All patients (N = 137)
Follow-up time, days (IQR)	661.0 (334.5–939.5)
Decompensation event, n (%)	57 (41.6%)
Ascitic complication, n (%)	20 (14.6%)
Variceal bleeding, n (%)	10 (7.3%)
Hepatic encephalopathy, n (%)	24 (17.5%)
ACLF, n (%)	33 (24.1%)
Acute kidney injury, n (%)	28 (20.4%)
Bacterial infection, n (%)	34 (24.8%)
HCC, n (%)	3 (2.2%)
TIPS implantation, n (%)	11 (8.0%)
Liver transplantation, n (%)	11 (8.0%)
Death, n (%)	32 (23.4%)
Liver-related death, n (%)	24 (17.5%)

ACLF, acute-on-chronic liver failure; HCC, hepatocellular carcinoma; TIPS, transjugular intrahepatic portosystemic shunt.

### Impact of t-Cort and f-Cort on clinical outcomes in patients with ACLD

Table 3 shows the impact of t-Cort on the risk of bacterial infections, decompensation/further decompensation, ACLF, and liver-related death. After adjustment for relevant cofactors, lower t-Cort was associated with a significantly higher risk of bacterial infections (asHR: 1.11; 95% CI: 1.04–1.19; *p* = 0.002), decompensation/further decompensation (asHR: 1.08; 95% CI: 1.02–1.12; *p* = 0.008), ACLF (asHR: 1.11; 95% CI: 1.04-1.19; *p* = 0.002) and liver-related death (asHR: 1.09; 95% CI: 1.01–1.18; *p* = 0.045).

**Table 3:** Impact of serum total cortisol on the risk of (i) bacterial infections, (ii) decompensation/further decompensation, (iii) acute-on-chronic liver failure (ACLF) and (iv) liver-related death.

Parameter of interest	Univariate (unadjusted) analysis			Multivariate (adjusted) analysis		
	**sHR**	**95% CI**	***p* value**	**asHR**	**95% CI**	***p* value**
**(i) Bacterial infections**						
Serum total cortisol, μg/dl∗	1.08	1.01–1.15	**0.022**	1.11	1.04–1.19	**0.002**
Age, 10 years	1.16	0.88–1.53	0.300	—	—	—
Sex (male)	1.14	0.52–2.50	0.740	—	—	—
Child Turcotte Pugh score, points	1.09	0.93–1.28	0.310	—	—	—
Creatinine, mg/dl	4.70	2.59–8.52	**<0.001**	4.55	2.57–8.07	**<0.001**
Sodium, mmol/L	0.99	0.88–1.14	0.970	—	—	—
HVPG, mmHg	1.02	0.97–1.08	0.420	—	—	—
C–reactive protein, mg/dl	1.29	1.02–1.64	**0.036**	1.26	0.98–1.61	0.072
**(ii) Decompensation/further decompensation**						
Serum total cortisol, μg/dl∗	1.06	1.01–1.12	**0.019**	1.08	1.02–1.12	**0.008**
Age, 10 years	0.88	0.69–1.13	0.330	—	—	—
Sex (male)	0.95	0.54–1.66	0.850	—	—	—
Child Turcotte Pugh score, points	1.30	1.16–1.47	**<0.001**	1.08	0.92–1.27	0.330
Creatinine, mg/dl	2.88	1.72–4.81	**<0.001**	2.19	1.12–4.26	**0.021**
Sodium, mmol/L	0.88	0.82–0.95	**<0.001**	0.90	0.83–0.98	**0.009**
HVPG, mmHg	1.13	1.08–1.18	**<0.001**	1.09	1.03–1.16	**0.003**
C–reactive protein, mg/dl	1.60	1.31–1.97	**<0.001**	1.30	1.03–1.67	**0.031**
**(iii) ACLF**						
Serum total cortisol, μg/dl∗	1.10	1.04–1.18	**0.001**	1.11	1.04–1.19	**0.002**
Age, 10 years	1.09	0.83–1.44	0.540	—	—	—
Sex (male)	1.26	0.58–2.75	0.560	—	—	—
Child Turcotte Pugh score, points	1.26	1.09–1.47	**0.002**	1.27	1.07–1.51	**0.005**
Creatinine, mg/dl	4.22	2.25–7.89	**<0.001**	4.03	2.12–7.64	**<0.001**
Sodium, mmol/L	0.95	0.85–1.06	0.320	—	—	—
HVPG, mmHg	1.03	0.98–1.09	0.220	—	—	—
C–reactive protein, mg/dl	1.23	0.95–1.58	0.110	—	—	—
**(iv) Liver–related death**						
Serum total cortisol, μg/dl∗	1.08	1.01.–1.15	**0.037**	1.09	1.01–1.18	**0.045**
Age, 10 years	1.10	0.78–1.55	0.590	—	—	—
Sex (male)	1.34	0.53–3.40	0.540	—	—	—
Child Turcotte Pugh score, points	1.28	1.09–1.51	**0.002**	1.25	1.03–1.52	**0.026**
Creatinine, mg/dl	3.20	1.29–7.96	**0.012**	3.03	1.23–7.42	**0.016**
Sodium, mmol/L	0.98	0.91–1.06	0.630	—	—	—
HVPG, mmHg	1.06	0.99–1.13	0.069	1.03	0.95–1.11	0.500
C–reactive protein, mg/dl	1.27	0.92–1.76	0.140	—	—	—

Univariate and multivariate multivariate competing risk regression models are shown. Liver transplantation and death were considered as competing risks for (i) and (iii), while liver transplantation and non-liver-related death were considered as competing risks for (ii) and (iv). Adjusted subdistribution hazard ratio (asHR) with 95% confidence interval (95% CI) and significance levels of multivariate Fine and Gray competing risk regression models for serum total cortisol: (i) asHR: 1.11 (95% CI: 1.04–1.19), *p* = 0.002; (ii) asHR: 1.08 (95% CI: 1.02–1.12), *p* = 0.008; (iii) asHR: 1.11 (95% CI: 1.04–1.19), *p* = 0.002; (iv) asHR: 1.09 (95% CI 1.01–1.18), *p* = 0.045. Statistically significant values are provided in bold.

∗ Indicated as a continuous variable from higher to lower serum total cortisol levels.

Interestingly, f-Cort performed comparably well in the prediction of clinical outcomes (Table S3). Lower f-Cort was associated with an increased risk of bacterial infections (asHR: 1.14; 95% CI: 1.02–1.25; *p* = 0.014), ACLF (asHR: 1.19; 95% CI: 1.06–1.33; *p* = 0.003), liver-related death (asHR: 1.14; 95% CI: 1.01–1.30; *p* = 0.041) and a non-significant higher risk of further decompensation (asHR: 1.08; 95% CI: 1.00–1.18; *p* = 0.054).

### t-Cort and f-Cort cut-off points for risk stratification in stable outpatients with ACLD

A t-Cort level of <12.0 μg/dl and an f-Cort level of <4.8 ng/ml were determined by the Youden index as the ideal cut-off points for the prediction of ACLF in our cohort of stable outpatients with ACLD. Overall, 63.5% (n = 87/137) of the patients had t-Cort levels <12 μg/dl and 46.6% (n = 62/133) of the patients had f-Cort <4.8 ng/ml.

As shown in Table S4, patients with t-Cort <12 μg/dl and t-Cort ≥12 μg/dl, as well as patients with f-Cort <4.8 ng/ml and f-Cort ≥4.8 ng/ml did not significantly differ in key parameters, but those with t-Cort <12 μg/dl had numerically more advanced ACLD (CTP score: <12 μg/dl: 7.0 points vs. ≥12 μg/dl: 6.0 points; *p* = 0.103) and more severe PH (HVPG: <12 μg/dl: 17 mmHg vs. ≥12 μg/dl: 15 mmHg; *p* = 0.239). Similarly, patients with f-Cort <4.8 ng/ml tended to have a higher clinical stage of ACLD (*p* = 0.121) and f-Cort <4.8 ng/ml was observed twice as frequently in patients with ACLD substage S5 compared with those with substage S0 (S5: 42.4% [n = 14/33] *vs.* S0: 18.2% [n = 2/11]).

Patients with t-Cort <12 μg/dl more frequently experienced further decompensation (50.6% *vs*. ≥12 μg/dl: 26.0%; *p* = 0.005, Table S5), ACLF (33.3% *vs*. ≥12 μg/dl: 8.0%; *p* <0.001), and liver-related death (23.0% *vs*. ≥12 μg/dl: 8.0%; *p* = 0.026). Moreover, the prevalence of bacterial infections was higher in the t-Cort <12 μg/dl grou*p* (33.3% *vs*. ≥12 μg/dl: 10.0%; *p* = 0.002). Similar results were obtained for patients with f-Cort <4.8 ng/ml.

**Fig. 5:**
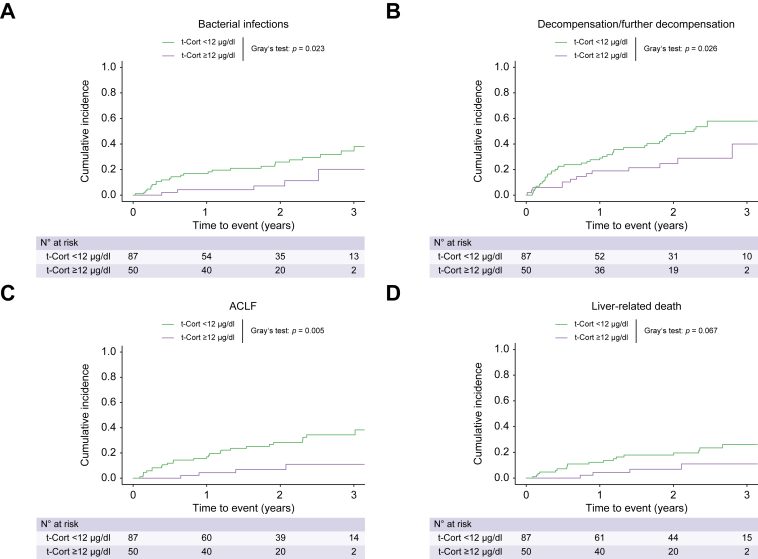
**Clinical outcomes**. Cumulative incidence of (A) bacterial infections, (B) first/further hepatic decompensation, (C) acute-on-chronic liver failure (ACLF) and (D) liver-related death within five years of follow-u*p* stratified by serum total cortisol (t-Cort) levels. (A, C) Liver transplantation and death and (B, D) liver transplantation and non-liver related death were considered competing risks, respectively. Levels of significance of cumulative incidences compared via Gray’s test: (A) *p* = 0.023; (B) *p* = 0.026; (C) *p* = 0.005; (D) *p* = 0.067.

As shown in Fig. 5, patients with t-Cort <12 μg/dl exhibited a higher cumulative incidence of bacterial infections (*p* = 0.023; Table S6), further decompensation (*p* = 0.026), ACLF (*p* = 0.005), and a tendency for more frequent liver-related death (*p* = 0.067). Similarly, patients with f-Cort <4.8 mg/ml had numerically higher cumulative incidences of bacterial infections (*p* = 0.131) (Fig. S1), further decompensation (*p* = 0.058), and ACLF (*p* = 0.104).

After adjustment for clinically relevant cofactors, t-Cort <12 μg/dl was independently associated with a higher risk of bacterial infections (asHR: 3.17; 95% CI: 1.24-8.09; *p* = 0.016; Table S7), further decompensation (asHR: 2.39; 95% CI: 1.31-4.35; *p* = 0.043), ACLF (asHR: 3.91; 95% CI: 1.36-11.29; *p* = 0.012), and in a tendency for more frequent liver-related death (asHR: 2.70; 95% CI: 0.91–8.02; *p* = 0.073). Similar results were found for patients with f-Cort 4.8 ng/ml (Table S8).

## Discussion

In this prospective exploratory study, we investigated the pituitary–adrenal axis in a thoroughly characterised cohort of stable outpatients with ACLD. We observed a decline in ACTH and t-Cort levels with the progressive decline in clinical stage of ACLD. Importantly, low levels of levels of t-Cort and f-Cort were independent risk factors for clinical events when analysed as continuous and dichotomised variables. This suggests that even in stable outpatients with ACLD, decreased levels of cortisol may be of clinical and prognostic relevance.

Dysregulation of cortisol metabolism and homeostasis have been described in patients with ACLD. These include reduced 11β-hydroxysteroid dehydrogenase type 2 activity with consecutively increased circulating glucocorticoids,^35^^,^^36^ decreased elimination rates of free cortisol, as well as reduced maximal cortisol secretion rates.^18^ Previous studies have mostly focused on adrenal function in patients with cirrhosis and acute decompensation and/or in those admitted to intensive care: Inadequate adrenal response, as assessed by a short synacthen test (SST), was often reported in acutely decompensated patients with cirrhosis. Relative adrenal insufficiency was observed in a significant share of patients (between 24% and 60% of cirrhotic patients)^17^^,^[37], [38], [39]^,^ and acutely hospitalised patients with relative adrenal insufficiency showed inferior clinical outcomes with decreased survival,^37^ and these patients more often experienced decompensation events, including bleeding and hepatorenal syndrome.^40^

However, the presence and clinical relevance of decreased t-Cort and f-Cort levels have not yet been specifically investigated in stable outpatients with ACLD. In this study, we found a decrease in t-Cort levels with the progressive severity of ACLD. It should be noted that 90% of circulating cortisol is bound to CBG and, to a lesser extent, to albumin, which are both produced in the liver and thus decreased in patients with ACLD.^20^^,^^41^ Accordingly, CBG levels decreased significantly throughout all clinical stages of ACLD in our study, and the resulting median levels of f-Cort were not significantly different between different stages of ACLD, suggesting a maintained homeostasis of cortisol in many patients with ACLD, even in more advanced clinical stages. This is in line with previous data showing an absence of dynamics of the biologically free serum cortisol fraction across patients in different CTP stages.^18^^,^^42^ Reduced elimination of f-Cort in patients with more severe ACLD^18^ may be involved in the largely maintained levels of f-Cort.

However, both low t-Cort and low f-Cort indicated an increased risk of subsequent clinical complications, especially bacterial infections and ACLF, possibly indicating altered cortisol secretion with an impaired stress response in a subgroup of patients with ACLD. Interestingly, low f-Cort and t-Cort are related to conditions of chronic inflammation.^23^ Low f-Cort and t-Cort are observed more frequently in patients with severe ACLD. Specifically, f-Cort <4.8 ng/ml was twice as prevalent in patients with ACLD substage S5 as in substage S0 (42.4% *vs.* 21.4%), whereas comparable findings were obtained for f-Cort <12 μg/dl (Fig. 1).

Notably, since t-Cort and f-Cort showed a strong correlation and similar prognostic utility, the assessment of t-Cort may be the preferable and more straight-forward surrogate marker for adrenal function in stable outpatients with ACLD without current infection or acute decompensation. Patients with t-Cort <12 μg/dl or f-Cort <4.8 ng/ml appear to be at particularly higher risk of subsequent development of bacterial infections and ACLF. Further studies are required to determine whether these patients may benefit from specific therapeutic interventions, such as antibiotic prophylaxis and/or closer clinical monitoring.

The observed decline in ACTH with progressing substages of ACLD is consistent with a previous study showing that direct stimulation of the adrenal gland through ACTH administration led to increased cortisol responses compared with indirect stimulation,^20^ whereas another study found frequent impairment of the pituitary–adrenal axis through the corticotropin releasing hormone (CRH) stimulation test, particularly in patients with CTP stage B and C.^43^ Hence, there is evidence that impaired adrenal function in ACLD is at least partially caused by a dysfunction of stimulating pituitary–adrenal signalling.

Bile acid signalling might have been a factor involved in the pathogenesis of the adrenal dysfunction observed in this study. Bile acids modify cortisol homeostasis by suppressing CRH excretion^21^ and influencing cortisol metabolism and catabolism through farnesoid X receptor (FXR) signalling.^44^ Furthermore, although acute inflammation is linked to activation of the hypothalamus–pituitary–adrenal axis, chronic inflammation seems to have an opposite effect, suppressing hypothalamus–pituitary–adrenal signalling.^23^ Indeed, our data showed an increase of both systemic inflammation (reflected by IL-6),^5^ as well as systemic bile acid levels with a more advanced clinical stage. These two factors may interfere with cortisol homeostasis and pituitary–adrenal signalling with increasing severity of ACLD.

Although previous research indicated that decreased levels of total and HDL cholesterol are involved in the impairment of adrenal function in ACLD,^17^^,^^45^ in our study, total and HDL cholesterol levels were not significantly different in different substages. This might be because, we included stable outpatients with mostly preserved liver function (*i.e.* primarily CTP stages A and B).

Our study also has limitations. First, we did not perform SST or sequential measurements in each individual patient. Thus, we cannot investigate relative adrenal insufficiency or SST responses in this study population. Second, the adequacy of t-Cort measurements has been debated in patients with cirrhosis for various reasons, including assay dependency^46^^,^^47^ or the lack of validated reference values specific to ACLD or hypoprotein-corrected reference values.^47^ Of note, our study found robust results for t-Cort and t-Cort also correlated significantly with f-Cort. However, we understand that external validation is required for the proposed f-Cort and t-Cort cut-offs at <4.8 ng/ml and <12 μg/dl, respectively. Moreover, the direct measurement of serum free cortisol is costly and complex, limiting its use in clinical practice. In this study, we used Coolen’s formula, a well-established and widely used formula to calculate f-Cort based on t-Cort and CBG. However, this formula has limitations, particularly in patient groups with expected variations of CBG and albumin. More refined equations adjusted for albumin levels may be more precise in estimating serum-free cortisol^48^ in patients with ACLD, but may also be more difficult to use in clinical practise. Notably, we did not include patients with regular night working hours, or individuals with undisclosed co-factors that may have impacted on circadian rhythm.

Our patients exhibited a relatively high median mean arterial pressure (MAP). This underlines that this study involved a stable patient population with ACLD without acute decompensation or critical illness and should be interpreted as such. As ALD was the main liver disease aetiology in our patient cohort and because chronic alcohol consumption influences cortisol homeostasis,^35^ it should be emphasised that most (*i.e.* 84.4%) of the included patients with ALD were abstinent at the time of characterisation.

Most patients in stage 4 and 5 ACLD were treated with diuretics, specifically aldosterone receptor antagonists that may have likely affected aldosterone levels.^49^^,^^50^ Finally, our observational study cannot establish a causative association between decreased levels of cortisol and adverse clinical outcomes. Thus, it is unclear whether decreased levels of cortisol are solely prognostic biomarkers or if they may serve as a therapeutic target.

In conclusion, our study demonstrates that pituitary ACTH and adrenal t-Cort levels decrease with progressive clinical substages of ACLD, suggesting suppression of the hypothalamic–pituitary–adrenal axis, particularly in patients with dACLD. Increased levels of systemic inflammation and bile acids might be associated with the suppression of pituitary–adrenal cortisol signalling. Importantly, low levels of t-Cort and low levels of f-Cort are associated with worse clinical outcomes and indicate a higher risk of subsequent development of bacterial infections, further decompensation, ACLF, and liver-related death. Additional studies are required to determine whether specific therapeutic interventions or closer clinical monitoring are warranted in patients with ACLD and low levels of f-Cort or t-Cort.

## Financial support

No specific funding was received for this study.

## Authors’ contributions

All authors contributed either to research design (LH and TR) and/or the acquisition (LH, BSim, MJ, DB, RP, BSch, LB, GS, MM, and TR), analysis (LH, PW, MM, and TR) or interpretation (all authors) of data. LH and TR drafted the manuscript, which was critically revised by all other authors.

## Data availability statement

The data derived from this study are available upon reasonable request to the corresponding author.

## Conflicts of interest

The authors have nothing to disclose regarding the work under consideration for publication. Conflicts of interests outside the submitted work: LH, MJ, PW, LB, GS, MS, and RM have nothing to disclose. BSim. received travel support from AbbVie and Gilead. DJMB received speaker fees from AbbVie and Siemens, as well as grant support form Gilead and Siemens, as well as travel support from AbbVie and Gilead. BSch. received travel support from AbbVie, Ipsen, and Gilead. MT served as a speaker and/or consultant and/or advisory board member for Albireo, BiomX, Falk, Boehringer Ingelheim, Bristol-Myers Squibb, Falk, Genfit, Gilead, Intercept, Janssen, MSD, Novartis, Phenex, Pliant, Regulus, and Shire, and received travel support from AbbVie, Falk, Gilead, and Intercept, as well as grants/research support from Albireo, Alnylam, Cymabay, Falk, Gilead, Intercept, MSD, Takeda, and UltraGenyx. He is also co-inventor of patents on the medical use of 24-norursodeoxycholic acid. MM served as a speaker and/or consultant and/or advisory board member for AbbVie, Collective Acumen, Gilead, Takeda, and W. L. Gore & Associates and received travel support from AbbVie and Gilead. TR served as a speaker and/or consultant and/or advisory board member for AbbVie, Bayer, Boehringer Ingelheim, Gilead, Intercept, MSD, Siemens, and W. L. Gore & Associates and received grants/research support from AbbVie, Boehringer Ingelheim, Gilead, Intercept, MSD, Myr Pharmaceuticals, Pliant, Philips, Siemens, and W. L. Gore & Associates as well as travel support from AbbVie, Boehringer Ingelheim, Gilead and Roche.

Please refer to the accompanying ICMJE disclosure forms for further details.
