# Supplementary material for: An impaired pituitary–adrenal signalling axis in stable cirrhosis is linked to worse prognosis
Source: JHEP Rep. 2023 May 11;5(8):100789. doi: 10.1016/j.jhepr.2023.100789 (PMC10362733; doi:10.1016/j.jhepr.2023.100789)
Supplement: Multimedia component 1 [file mmc1.pdf]

Supplementary material

An impaired pituitary–adrenal signalling axis in stable cirrhosis is linked to worse prognosis

Lukas Hartl[1,2], Benedikt Simbrunner[1,2,3], Mathias Jachs[1,2], Peter Wolf[4], David Josef Maria Bauer[1,2], Bernhard Scheiner[1,2], Lorenz Balcar[1,2], Georg Semmler[1,2], Michael Schwarz[1], Rodrig Marculescu[5], Michael Trauner[1], Mattias Mandorfer[1,2], Thomas Reiberger

Table of contents

Fig. S1.	Page 2
Table S1.	Page 3
Table S2.	Page 5
Table S3.	Page 6
Table S4.	Page 8
Table S5.	Page 11
Table S6.	Page 13
Table S7.	Page 14
Table S8.	Page 16

FIGURES

Figure-S1. Cumulative incidence of (A) bacterial infections, (B) first/further hepatic decompensation, (C) acute-on-chronic liver failure (ACLF) and (D) liver-related death within five years of follow-up stratified by serum free cortisol (f-Cort) levels. (A, C) Liver transplantation and death and (B, D) liver transplantation and non-liver related death were considered competing risks, respectively. Levels of significance of cumulative incidences compared via Gray's test: (A) p=0.131; (B) p=0.058; (C) p=0.104; (D) p=0.326.

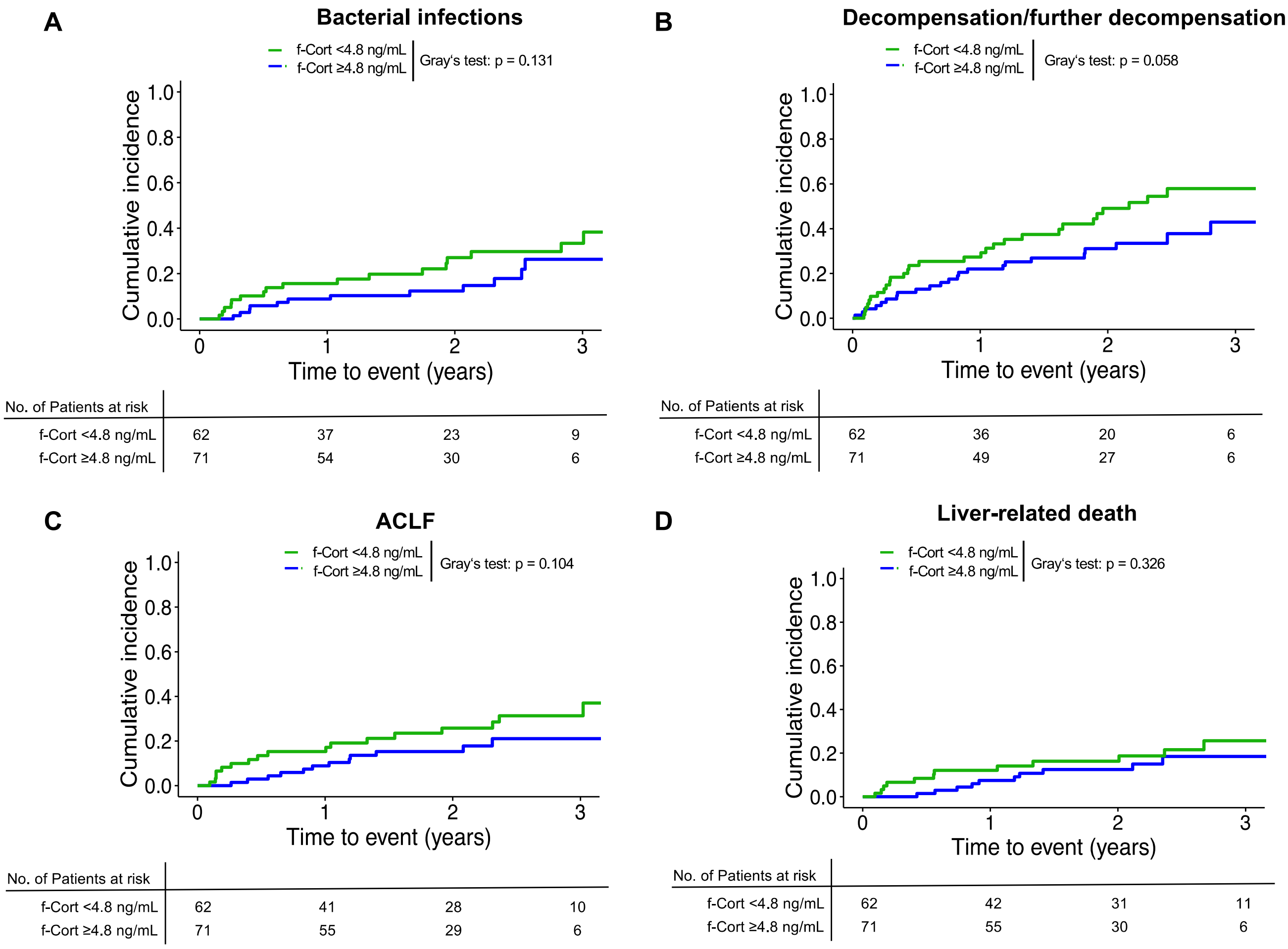

TABLES

Table-S1. ACTH-cortisol axis, aldosterone, cholesterol levels and liver function parameters in patients with different stages of advanced chronic liver disease. Significance level of group comparisons via Kruskal-Wallis test: ACTH: p=0.006; serum total cortisol: p=0.091; cortisol binding globulin: p<0.001; serum free cortisol: p=0.474; serum total cortisol/serum free cortisol ratio: p=0.002; corticosterone: p=0.826; aldosterone: p<0.001; CTP score: p<0.001; MELD: p<0.001; albumin: p<0.001; bile acids: p<0.001; total cholesterol: p=0.818; HDL cholesterol: p=0.392; IL-6: p<0.001.

**Parameter of interest**	**EASL stage**						**p-value**
	**0 (n=13)**	**1 (n=12)**	**2 (n=26)**	**3 (n=7)**	**4 (n=46)**	**5 (n=33)**	
**ACTH, pg x mL^{1} (IQR)**	44.0 (29.0-123.5)	22.5 (13.0-42.0)	21.0 (11.5-29.8)	25.0 (15.0-35.0)	19.0 (9.0-25.0)	20.0 (11.0-31.5)	**0.006**
**Serum total cortisol, µg x dL^{-1} (IQR)**	13.9 (10.6-20.4)	12.0 (6.1-13.5)	8.7 (5.7-13.9)	11.1 (10.0-13.4)	8.6 (5.9-12.1)	9.2 (6.1-13.1)	0.091
**Cortisol binding globulin, µg x mL^{1} (IQR)[2]**	49.3 (41.7-58.7)	48.6 (38.8-60.4)	43.8 (38.0-51.6)	43.4 (37.6-50.7)	36.6 (32.3-43.5)	38.9 (30.4-44.9)	**<0.001**
**Serum free cortisol, ng x mL^{-1} (IQR)[2]**	6.5 (4.8-8.8)	5.0 (2.5-6.7)	3.6 (2.4-6.8)	6.3 (4.5-6.8)	4.6 (3.0-7.7)	5.7 (3.8-7.5)	0.474
**Serum total cortisol/serum free cortisol ratio, median (IQR)[2]**	21.2 (16.7-31.8)	22.5 (18.7-27.1)	22.2 (16.6-27.4)	20.0 (16.9-22.3)	17.7 (14.4-21.8)	18.2 (13.8-21.2)	0.002
**Corticosterone, ng x mL^{-1} (IQR)[1]**	5.7 (3.2-23.5)	6.0 (3.7-10.4)	6.0 (3.0-9.2)	8.2 (8.1-8.3)	6.2 (4.5-13.9)	6.7 (4.9-14.1)	0.826
**Aldosterone, pg x mL^{-1} (IQR) [3]**	78.0 (62.5-140.5)	91.0 (49.0-110.0)	59.0 (44.5-87.8)	49.0 (38.0-81.0)	280.0 (156.3-496.3)	262.0 (86.5-604.0)	**<0.001**

**CTP score, points (IQR)**	5.0 (5.0-5.0)	5.0 (5.0-5.8)	5.0 (5.0-6.0)	5.0 (5.0-5.0)	7.0 (6.0-8.3)	8.0 (7.0-9.0)	**<0.001**
**MELD, median (IQR)**	8.0 (6.5-11.5)	9.0 (8.3-14.5)	10.0 (9.0-11.0)	10.0 (8.0-12.0)	13.0 (11.0-17.0)	14.0 (11.0-16.0)	**<0.001**
**Albumin, g x L^{-1} (IQR)**	41.9 (40.0-44.2)	39.7 (35.2-40.6)	38.4 (36.5-40.8)	40.7 (39.2-41.1)	35.5 (32.4-38.4)	35.4 (28.2-37.0)	**<0.001**
**Bile acids, µmol x L^{-1} (IQR)**	2.7 (1.7-6.1)	9.3 (5.8-13.5)	12.3 (8.9-31.2)	6.3 (3.9-14.8)	18.5 (10.2-46.3)	36.5 (11.7-70.9)	**<0.001**
**Total cholesterol, mg x dL^{-1} (IQR)**	149.0 (126.5-176.5)	136.5 (107.5-181.8)	147.0 (122.8-168.5)	138.0 (119.0-149.0)	139.5 (109.8-168.3)	133.0 (117.0-153.0)	0.818
**HDL cholesterol, mg x dL^{-1} (IQR)**	41.0 (37.0-53.0)	43.5 (33.8-49.8)	50.5 (38.3-64.5)	47.0 (41.0-56.0)	38.0 (32.0-55.0)	47.0 (32.5-57.5)	0.392
**IL-6, ng x dL^{-1} (IQR)**	4.7 (3.0-8.6)	7.4 (3.9-15.4)	4.8 (4.4-8.4)	3.1 (2.8-5.0)	10.4 (5.9-21.5)	10.8 (6.4-25.0)	**<0.001**

[1] available in 133 patients (stage 0: n=11; stage 1: n=12; stage 2: n=26; stage 3: n=6; stage 4: n=45; stage 5: n=33)

[2] available in 77 patients (stage 0: n=6; stage 1: n=5; stage 2: n=17; stage 3: n=2; stage 4: n=25; stage 5: n=22)

[3] available in 136 patients (stage 0: n=13; stage 1: n=11; stage 2: n=26; stage 3: n=7; stage 4: n=46; stage 5: n=33)

Table-S2. ACTH-cortisol axis, aldosterone and cholesterol levels in patients with compensated (cACLD) and decompensated advanced chronic liver disease (dACLD). Significance level of group comparisons via Mann Whitney U test: ACTH: p=0.193; serum total cortisol: p=0.260; cortisol binding globulin: p<0.001; serum free cortisol: p=0.872; corticosterone: p=0.354; aldosterone: p<0.001; bile acids: p<0.001; total cholesterol: p=0.818; HDL cholesterol: p=0.392; LDL cholesterol: p=0.876.

**Parameter**	**cACLD (n=51)**	**dACLD (n=86)**	**p-value**
**ACTH, pg x mL[1] (IQR)**	24.0 (15.0-43.0)	20.0 (10.8-26.0)	0.193
**Serum total cortisol, µg x dL^{-1} (IQR)**	11.0 (6.3-15.1)	9.3 (6.0-12.3)	0.260
**Cortisol binding globulin, µg x mL^{-1} (IQR) [1]**	44.6 (39.5-53.7)	38.8 (32.2-44.4)	**<0.001**
**Serum free cortisol, ng x mL^{-1} (IQR) [1]**	4.8 (2.5-6.9)	5.3 (3.2-7.5)	0.872
**Corticosterone, ng x mL^{-1} (IQR) [2]**	6.0 (3.4-9.8)	6.9 (4.8-13.8)	0.354
**Aldosterone, pg x mL^{-1} (IQR) [3]**	72.0 (48.8-100.0)	261.0 (107.8-496.3)	**<0.001**
**Bile acids, µmol x L^{-1} (IQR)**	9.4 (5.1-17.7)	22.1 (10.1-50.5)	**<0.001**
**Total cholesterol, mg x dL^{-1} (IQR)**	146.0 (123.0-170.0)	137.0 (114.8-165.5)	0.582
**HDL cholesterol, mg x dL^{-1} (IQR)**	46.0 (37.0-54.0)	42.5 (32.8-42.5)	0.582
**LDL cholesterol, mg x dL^{-1} (IQR)**	77.2 (59.2-105.2)	76.9 (57.6-100.7)	0.876

[1] available in n=133 patients (cACLD: n=49, dACLD: n=84)

[2] available in n=77 patients (cACLD: n=28, dACLD: n=49)

[3] available in 136 patients (cACLD: n=50, dACLD: n=86)

Table-S3. Impact of serum free cortisol on the risk of (i) bacterial infections, (ii) decompensation/further decompensation, (iii) acute-on-chronic liver failure (ACLF) and (iv) liver-related death. Univariate and multivariate multivariate competing risk regression models are shown. Liver transplantation and death were considered as competing risks for (i) and (iii), while liver transplantation and non-liver-related death were considered as competing risks for (ii) and (iv). Adjusted subdistribution hazard ratio (asHR) with 95% confidence interval (95%CI) and significance levels of multivariate Fine and Gray competing risk regression models for serum total cortisol: (i) asHR: 1.14 (95%CI 1.02-1.25), p=0.014; (ii) asHR: 1.08 (95%CI 1.00-1.18), p=0.054; (iii) asHR: 1.19 (95%CI 1.06-1.33), p=0.003; (iv) asHR: 1.14 (95%CI 1.01-1.30), p=0.041.

**Parameter of interest**	**Univariate (unadjusted) analysis**			**Multivariate (adjusted) analysis**		
**(i) bacterial infections**	**sHR**	**95%CI**	**p-value**	**asHR**	**95%CI**	**p-value**
**Serum free cortisol, ng x mL^{-1} ***	1.08	0.98-1.18	0.110	1.14	1.02-1.25	**0.014**
**Age, 10 years**	1.16	0.88-1.53	0.300	-	-	-
**Sex (male)**	1.14	0.52-2.50	0.740	-	-	-
**Child Turcotte Pugh score, points**	1.09	0.93-1.28	0.310	-	-	-
**Creatinine, mg x dL^{-1}**	4.70	2.59-8.52	**<0.001**	4.82	2.45-9.46	**<0.001**
**Sodium, mmol x L^{-1}**	0.99	0.88-1.14	0.970	-	-	-
**HVPG, mmHg**	1.02	0.97-1.08	0.420	-	-	-
**C-reactive protein, mg x dL^{-1}**	1.29	1.02-1.64	**0.036**	1.31	1.03-1.66	**0.028**
**(ii) decompensation/further decompensation**						
**Serum free cortisol, ng x mL^{-1} ***	1.02	0.93-1.12	0.690	1.08	1.00-1.18	0.054
**Age, 10 years**	0.88	0.69-1.13	0.330	-	-	-
**Sex (male)**	0.95	0.54-1.66	0.850	-	-	-
**Child Turcotte Pugh score, points**	1.30	1.16-1.47	**<0.001**	1.16	1.00-1.36	0.051
**Creatinine, mg x dL^{-1}**	2.88	1.72-4.81	**<0.001**	2.21	1.11-4.39	**0.024**

**Sodium, mmol x L^{-1}**	0.88	0.82-0.95	**<0.001**	0.90	0.83-0.98	**0.012**
**HVPG, mmHg**	1.13	1.08-1.18	**<0.001**	1.09	0.83-0.98	**0.008**
**C-reactive protein, mg x dL^{-1}**	1.60	1.31-1.97	**<0.001**	1.35	1.06-1.72	**0.015**
**(iii) ACLF**	**sHR**	**95%CI**	**p-value**	**asHR**	**95%CI**	**p-value**
**Serum free cortisol, ng x mL^{-1} ***	1.10	1.01-1.20	**0.021**	1.19	1.06-1.33	**0.003**
**Age, 10 years**	1.09	0.83-1.44	0.540	-	-	-
**Sex (male)**	1.26	0.58-2.75	0.560	-	-	-
**Child Turcotte Pugh score, points**	1.26	1.09-1.47	**0.002**	1.42	1.16-1.74	**<0.001**
**Creatinine, mg x dL^{-1}**	4.22	2.25-7.89	**<0.001**	3.98	1.98-8.01	**<0.001**
**Sodium, mmol x L^{-1}**	0.95	0.85-1.06	0.320	-	-	-
**HVPG, mmHg**	1.03	0.98-1.09	0.220	-	-	-
**C-reactive protein, mg x dL^{-1}**	1.23	0.95-1.58	0.110	-	-	-
**(iv) Liver-related death**						
**Serum free cortisol, ng x mL^{-1} ***	1.06	0.97-1.15	0.170	1.14	1.01-1.30	**0.041**
**Age, 10 years**	1.10	0.78-1.55	0.590	-	-	-
**Sex (male)**	1.34	0.53-3.40	0.540	-	-	-
**Child Turcotte Pugh score, points**	1.28	1.09-1.51	**0.002**	1.32	1.06-1.65	**0.014**
**Creatinine, mg x dL^{-1}**	3.20	1.29-7.96	**0.012**	3.11	1.26-7.66	**0.014**
**Sodium, mmol x L^{-1}**	0.98	0.91-1.06	0.630	-	-	-
**HVPG, mmHg**	1.06	0.99-1.13	0.069	1.03	0.95-1.12	0.430
**C-reactive protein, mg x dL^{-1}**	1.27	0.92-1.76	0.140	-	-	-

*** indicated as a continuous variable from higher to lower f-Cort levels**

Table-S4. Patient characteristics according to low vs. high serum total cortisol (t-Cort) and serum free cortisol (f-Cort) levels[1].

Levels of significance of cumulative incidences compared via Chi squared test: Sex: t-Cort: p=0.461/f-Cort: p=0.468; etiology: t-Cort: p=0.268/f-Cort: p=0.140; decompensated ACLD: t-Cort: p=0.214/f-Cort: p=0.677; ascites: t-Cort: p=0.452/f-Cort: p=0.880; EASL stage: t-Cort: p=0.331/f-Cort: p=0.121; CTP stage: t-Cort: p=0.109/f-Cort: p=0.269; statin intake: t-Cort: p=0.511/f-Cort: p=0.520; Levels of significance of cumulative incidences compared via Mann Whitney U test: Age: t-Cort: p=0.097/f-Cort: p=0.012; BMI: t-Cort: p=0.910/f-Cort: p=0.926; MELD: t-Cort: p=0.639/f-Cort: p=0.572; CTP score: t-Cort: p=0.103/f-Cort: p=0.945; bilirubin: t-Cort: p=0.910/f-Cort: p=0.431; albumin: t-Cort: p=0.341/f-Cort: p=0.945; INR: t-Cort: p=0.094/f-Cort: p=0.889; sodium: t-Cort: p=0.661/f-Cort: p=0.573; HVPG: t-Cort: p=0.239/f-Cort: p=0.547; MAP: t-Cort: p=0.201/f-Cort: p=0.007; LSM: t-Cort: p=0.208/f-Cort: p=0.306; serum total cortisol: f-Cort: p<0.001; cortisol binding globulin: t-Cort: p<0.001/f-Cort: p=0.926; serum free cortisol: t-Cort: p<0.001; bile acids: t-Cort: p=0.158/f-Cort: p=0.907; total cholesterol: t-Cort: p=0.710/f-Cort: p=0.945; HDL cholesterol: t-Cort: p=0.987/f-Cort: p=0.444; LDL cholesterol: t-Cort: p=0.467/f-Cort: p=0.926; IL-6: t-Cort: p=0.640/f-Cort: p=0.660; WBC: t-Cort: p=0.594/f-Cort: p=0.862; CRP: t-Cort: p=0.859/f-Cort: p=0.851.

**Patient characteristics**	**Patients with t-Cort <12 µg/dL (n=87)**	**Patients with t-Cort ≥12 µg/dL (n=50)**	**p-value**	**Patients with f-Cort <4.8 µg/dL (n=62)**	**Patients with f-Cort ≥4.8 µg/dL (n=71)**	**p-value**
**Sex, male/female (% male)**	61/26 (70.1%)	32/18 (64.0%)	0.461	40/22 (64.5%)	50/21 (70.4%)	0.468
**Age, years (IQR)**	57.1 (49.4-64.4)	61.2 (51.2-68.3)	0.097	55.0 (48.0-61.5)	61.1 (51.4-68.6)	0.012
**BMI, kg x m^{-2} (IQR)**	25.9 (23.5-29.4)	26.1 (23.4-29.9)	0.910	26.0 (23.4-30.4)	26.0 (23.6-28.7)	0.926
**Etiology**			0.268			0.140

ALD, n (%)	44 (50.6%)	21 (42.0%)		29 (46.8%)	34 (47.9%)	
Viral hepatitis, n (%)	10 (11.5%)	12 (20.0%)		7 (11.3%)	12 (16.9%)	
ALD+Viral hepatitis, n (%)	10 (11.5%)	2 (4.0%)		9 (14.5%)	3 (4.2%)	
NASH, n (%)	11 (12.6%)	6 (12.0%)		9 (14.5%)	7 (9.9%)	
Cholestatic, n (%)	1 (1.1%)	0 (0.0%)		1 (1.6%)	0 (0.0%)	
Other, n (%)	11 (12.6%)	11 (22.0%)		7 (11.3%)	15 (21.1%)	
Decompensated ACLD, n (%)	58 (66.7%)	28 (56.0%)	0.214	38 (61.3%)	46 (64.8%)	0.677
Ascites, n (%)	51 (58.6%)	26 (52.0%)	0.452	35 (56.5%)	41 (57.7%)	0.880
EASL stage			0.331			0.121
0, n (%)	5 (5.7%)	8 (16.0%)		2 (3.2%)	9 (12.7%)	
1, n (%)	6 (6.9%)	6 (12.0%)		6 (9.7%)	6 (8.5%)	
2, n (%)	18 (20.7%)	8 (16.0%)		16 (25.8%)	10 (14.1%)	
3, n (%)	5 (5.7%)	2 (4.0%)		1 (1.6%)	5 (7.0%)	
4, n (%)	32 (36.8%)	14 (28.0%)		23 (37.1%)	22 (31.0%)	
5, n (%)	21 (24.2%)	12 (24.0%)		14 (22.6%)	19 (26.7%)	
MELD, points (IQR)	12.0 (9.0-16.0)	11.5 (8.0-14.0)	0.639	11.0 (9.0-14.3)	12.0 (9.0-16.0)	0.572
CTP score, points (IQR)	7.0 (5.0-8.0)	6.0 (5.0-7.3)	0.103	6.0 (5.0-7.3)	6.0 (5.0-8.0)	0.945
CTP stage			0.109			0.269
A, n (%)	46 (48.4%)	37 (66.1%)		37 (53.6%)	42 (54.5%)	
B, n (%)	39 (41.1%)	15 (26.8%)		28 (40.6%)	25 (32.5%)	
C, n (%)	10 (10.5%)	4 (7.1%)		4 (5.8%)	10 (13.0%)	
Bilirubin, mg x dL^{-1} (IQR)	1.2 (0.8-1.9)	1.1 (0.6-1.9)	0.910	1.1 (0.8-1.7)	1.3 (0.6-1.9)	0.431
Albumin, g x dL^{-1} (IQR)	36.9 (32.9-39.1)	39.0 (35.0-40.9)	0.341	37.0 (34.1-39.5)	37.0 (32.3-40.6)	0.945
INR, units (IQR)	1.4 (1.2-1.6)	1.3 (1.2-1.4)	0.094	1.4 (1.2-1.5)	1.4 (1.3-1.6)	0.889
Sodium, mmol x L^{-1} (IQR)	139.0 (137.0-141.0)	138.5 (136.0-140.0)	0.661	139.0 (137.0-142.0)	138.0 (136.0-140.0)	0.537
HVPG, mmHg (IQR)	17 (11-20)	15 (11-19)	0.239	17 (11-20)	16 (11-20)	0.547

**MAP, mmHg (IQR)**	99 (87-109)	104 (96-117)	0.201	95 (87-110)	104 (96-110)	0.007
**LSM, kPa (IQR)**	39.7 (21.6-61.8)	31.3 (20.1-49.4)	0.208	39.6 (22.6-65.2)	32.9 (18.6-50.3)	0.306
**Serum total cortisol, µg x dL^{-1} (IQR)**	-	-	-	6.0 (4.3-7.2)	13.0 (11.0-16.0)	<0.001
**Cortisol binding globulin, µg x mL^{-1} (IQR) [2]**	38.2 (33.1-44.3)	44.7 (40.0-53.4)	**<0.001**	40.7 (34.8-46.6)	41.7 (34.3-49.3)	0.926
**Serum free cortisol, ng x mL^{-1} (IQR) [2]**	3.5 (2.2-5.4)	7.9 (6.5-11.0)	**<0.001**	-	-	-
**Bile acids, µmol x L^{-1} (IQR)**	17.2 (6.2-46.6)	11.6 (6.8-26.4)	0.158	14.2 (6.5-40.3)	13.9 (6.8-42.4)	0.907
**Total cholesterol, mg x dL^{-1} (IQR)**	136.0 (115.0-168.0)	141.0 (123.5-167.5)	0.710	141.0 (117.8-168.3)	139.0 (115.0-167.0)	0.945
**HDL cholesterol, mg x dL^{-1} (IQR)**	44.0 (33.0-55.0)	43.5 (37.0-56.8)	0.987	46.5 (31.5-54.3)	43.0 (36.0-56.0)	0.444
**LDL cholesterol, mg x dL^{-1} (IQR)**	74.8 (56.2-98.2)	79.5 (59.2-103.2)	0.467	76.2 (58.0-101.6)	77.2 (58.0-100.6)	0.926
**Statin intake, n (%)**	12 (13.8%)	9 (18.0%)	0.511	8 (12.9%)	12 (16.9%)	0.520
**IL-6, ng x dL^{-1} (IQR)**	8.2 (4.2-15.6)	6.9 (3.7-13.0)	0.640	7.8 (3.9-15.1)	8.4 (4.4-16.8)	0.660
**WBC, G x L^{-1} (IQR)**	4.8 (3.4-5.9)	5.1 (4.0-6.5)	0.594	4.9 (3.5-6.0)	4.8 (3.7-6.4)	0.862
**CRP, mg x dL^{-1} (IQR)**	0.3 (0.1-0.8)	0.3 (0.1-0.8)	0.859	0.3 (0.1-0.8)	0.3 (0.1-1.0)	0.851

[1] f-Cort available in n=133 patients; t-Cort available in all patients (n=137);

Table-S5. Clinical outcomes of patients according to low vs. high serum total cortisol (t-Cort) and serum free cortisol (f-Cort) levels[1]. Levels of significance of cumulative incidences compared via Chi squared test: Decompensation event: t-Cort: p=0.005/f-Cort: p=0.039; ascitic complication: t-Cort: p=0.880/f-Cort: p=0.414; variceal bleeding: t-Cort: p=0.658/f-Cort: p=0.212; hepatic encephalopathy: t-Cort: p=0.198/f-Cort: p=0.413; ACLF: t-Cort: p<0.001/f-Cort: p=0.039; acute kidney injury: t-Cort: p=0.329/f-Cort: p=0.493; bacterial infection: t-Cort: p=0.002/f-Cort: p=0.063; HCC: t-Cort: p=0.184/f-Cort: p=0.061; TIPS implantation: t-Cort: p=0.496/f-Cort: p=0.958; liver transplantation: t-Cort: p=0.992/f-Cort: p=0.663; death: t-Cort: p=0.261/f-Cort: p=0.397; liver-related death: t-Cort: p=0.026/f-Cort: p=0.204. Levels of significance of cumulative incidences compared via Mann Whitney U test: Follow-up time: t-Cort: p=0.640/f-Cort: p=0.547.

**Clinical outcomes**	**Patients with t-Cort <12 µg/dL (n=87)**	**Patients with t-Cort ≥12 µg/dL (n=50)**	**p-value**	**Patients with f-Cort <4.8 µg/dL (n=62)**	**Patients with f-Cort ≥4.8 µg/dL (n=71)**	**p-value**
**Follow-up time, days (IQR)**	733.0 (277.0-1018.0)	624.5 (422.3-844,3)	0.640	717.5 (232.0-1007.5)	613.0 (413.0-871.0)	0.547
**Decompensation event, n (%)**	44 (50.6%)	13 (26.0%)	**0.005**	31 (50.0%)	23 (32.4%)	**0.039**
**Ascitic complication, n (%)**	13 (14.9%)	7 (14.0%)	0.880	10 (16.1%)	8 (11.3%)	0.414
**Variceal bleeding, n (%)**	7 (8.0%)	3 (6.0%)	0.658	6 (9.7%)	3 (4.2%)	0.212
**Hepatic encephalopathy, n (%)**	18 (20.7%)	6 (12.0%)	0.198	13 (21.0%)	11 (15.5%)	0.413
**ACLF, n (%)**	29 (33.3%)	4 (8.0%)	**<0.001**	20 (32.3%)	12 (16.9%)	**0.039**
**Acute kidney injury, n (%)**	20 (23.0%)	8 (16.0%)	0.329	11 (17.7%)	16 (22.5%)	0.493
**Bacterial infection, n (%)**	29 (33.3%)	5 (10.0%)	**0.002**	20 (32.3%)	13 (18.3%)	0.063
**HCC, n (%)**	3 (3.4%)	0 (0.0%)	0.184	3 (4.8%)	0 (0.0%)	0.061

**TIPS implantation, n (%)**	8 (9.3%)	3 (6.0%)	0.496	5 (8.2%)	6 (8.5%)	0.958
**Liver transplantation, n (%)**	7 (8.0%)	4 (8.0%)	0.992	4 (6.5%)	6 (8.5%)	0.663
**Death, n (%)**	23 (26.4%)	9 (18.0%)	0.261	17 (27.4%)	15 (21.1%)	0.397
**Liver-related death, n (%)**	20 (23.0%)	4 (8.0%)	**0.026**	14 (22.6%)	10 (14.1%)	0.204

[1] f-Cort available in n=133 patients; t-Cort available in all patients (n=137);

Table-S6. Cumulative incidences of clinical events in patients with ACLD and low vs. high serum total cortisol (t-Cort) and serum free cortisol (f-Cort) levels[1]. Levels of significance of cumulative incidences compared via Gray's test: (A) t-Cort: p=0.023/f-Cort: p=0.131; (B) t-Cort: p=0.026/f-Cort: p=0.058; (C) t-Cort: p=0.005/f-Cort: p=0.104; (D) t-Cort: p=0.067/f-Cort: p=0.326.

Clinical outcomes	Parameter	1 year	2 years	3 years	p-value
**(A) Bacterial infections**	t-Cort <12 µg/dL	16.9%	25.9%	34.6%	**0.023**
	t-Cort ≥12 µg/dL	4.3%	9.3%	20.2%	
	f-Cort <4.8 ng/mL	15.6%	27.0%	33.4%	0.131
	f-Cort ≥4.8 ng/mL	8.8%	12.3%	26.3%	
**(B) Decompensation/further decompensation**	t-Cort <12 µg/dL	27.7%	48.1%	57.9%	**0.026**
	t-Cort ≥12 µg/dL	19.0%	24.7%	40.0%	
	f-Cort <4.8 ng/mL	27.3%	49.1%	57.9%	0.058
	f-Cort ≥4.8 ng/mL	22.0%	31.1%	42.9%	
**(C) ACLF**	t-Cort <12 µg/dL	15.6%	28.4%	34.4%	**0.005**
	t-Cort ≥12 µg/dL	4.4%	6.9%	11.0%	
	f-Cort <4.8 ng/mL	15.2%	25.8%	31.3%	0.104
	f-Cort ≥4.8 ng/mL	8.9%	15.3%	21.0%	
**(D) Liver-related death**	t-Cort <12 µg/dL	12.2%	17.9%	26.1%	0.067
	t-Cort ≥12 µg/dL	4.4%	6.9%	10.9%	
	f-Cort <4.8 ng/mL	12.1%	16.2%	25.7%	0.326
	f-Cort ≥4.8 ng/mL	7.5%	12.5%	18.5%	

[1] f-Cort available in n=133 patients; t-Cort available in all patients (n=137);

Table-S7. Impact of serum total cortisol <12 µg/dL on the risk of (i) bacterial infections, (ii) decompensation/further decompensation, (iii) acute-on-chronic liver failure (ACLF) and (iv) liver-related death. Univariate and multivariate multivariate competing risk regression models are shown. Liver transplantation and death were considered as competing risks for (i) and (iii), while liver transplantation and non-liver-related death were considered as competing risks for (ii) and (iv). Adjusted subdistribution hazard ratio (asHR) with 95% confidence interval (95%CI) and significance levels of multivariate Fine and Gray competing risk regression models for serum total cortisol: (i) asHR: 3.17 (95%CI 1.24-8.09), p=0.016; (ii) asHR: 2.39 (95%CI 1.31-4.35), p=0.043; (iii) asHR: 3.91 (95%CI 1.36-11.29), p=0.012; (iv) asHR: 2.70 (95%CI 0.91-8.02), p=0.073.

**Parameter of interest**	**Univariate (unadjusted) analysis**			**Multivariate (adjusted) analysis**		
**(i) bacterial infections**	**sHR**	**95%CI**	**p-value**	**asHR**	**95%CI**	**p-value**
**Serum total cortisol <12 µg/dL, yes/no**	3.00	1.17-7.70	**0.022**	3.17	1.24-8.09	**0.016**
**Age, 10 years**	1.16	0.88-1.53	0.300	-	-	-
**Sex (male)**	1.14	0.52-2.50	0.740	-	-	-
**Child Turcotte Pugh score, points**	1.09	0.93-1.28	0.310	-	-	-
**Creatinine, mg x dL^{-1}**	4.70	2.59-8.52	**<0.001**	3.90	2.20-6.92	**<0.001**
**Sodium, mmol x L^{-1}**	0.99	0.88-1.14	0.970	-	-	-
**HVPG, mmHg**	1.02	0.97-1.08	0.420	-	-	-
**C-reactive protein, mg x dL^{-1}**	1.29	1.02-1.64	**0.036**	1.27	1.01-1.61	**0.045**
**(ii) decompensation/further decompensation**						
**Serum total cortisol <12 µg/dL, yes/no**	2.00	1.08-3.70	**0.027**	2.39	1.31-4.35	**0.043**
**Age, 10 years**	0.88	0.69-1.13	0.330	-	-	-
**Sex (male)**	0.95	0.54-1.66	0.850	-	-	-

**Child Turcotte Pugh score, points**	1.30	1.16-1.47	**<0.001**	1.06	0.90-1.25	0.470
**Creatinine, mg x dL^{-1}**	2.88	1.72-4.81	**<0.001**	1.95	1.01-3.76	**0.047**
**Sodium, mmol x L^{-1}**	0.88	0.82-0.95	**<0.001**	0.90	0.82-0.98	**0.012**
**HVPG, mmHg**	1.13	1.08-1.18	**<0.001**	1.09	1.03-1.17	**0.005**
**C-reactive protein, mg x dL^{-1}**	1.60	1.31-1.97	**<0.001**	1.36	1.11-1.67	**0.004**
**(iii) ACLF**	**sHR**	**95%CI**	**p-value**	**asHR**	**95%CI**	**p-value**
**Serum total cortisol <12 µg/dL, yes/no**	4.11	1.47-11.5	**0.007**	3.91	1.36-11.29	**0.012**
**Age, 10 years**	1.09	0.83-1.44	0.540	-	-	-
**Sex (male)**	1.26	0.58-2.75	0.560	-	-	-
**Child Turcotte Pugh score, points**	1.26	1.09-1.47	**0.002**	1.27	1.07-1.51	**0.005**
**Creatinine, mg x dL^{-1}**	4.22	2.25-7.89	**<0.001**	3.57	1.88-6.76	**<0.001**
**Sodium, mmol x L^{-1}**	0.95	0.85-1.06	0.320	-	-	-
**HVPG, mmHg**	1.03	0.98-1.09	0.220	-	-	-
**C-reactive protein, mg x dL^{-1}**	1.23	0.95-1.58	0.110	-	-	-
**(iv) Liver-related death**						
**Serum total cortisol <12 µg/dL, yes/no**	2.74	0.96-7.87	0.060	2.70	0.91-8.02	0.073
**Age, 10 years**	1.10	0.78-1.55	0.590	-	-	-
**Sex (male)**	1.34	0.53-3.40	0.540	-	-	-
**Child Turcotte Pugh score, points**	1.28	1.09-1.51	**0.002**	1.25	1.02-1.52	**0.030**
**Creatinine, mg x dL^{-1}**	3.20	1.29-7.96	**0.012**	2.68	1.12-6.44	**0.027**
**Sodium, mmol x L^{-1}**	0.98	0.91-1.06	0.630	-	-	-
**HVPG, mmHg**	1.06	0.99-1.13	0.069	1.03	0.95-1.10	0.480
**C-reactive protein, mg x dL^{-1}**	1.27	0.92-1.76	0.140	-	-	-

Table-S8. Impact of serum free cortisol <4.8 ng/mL on the risk of (i) bacterial infections, (ii) decompensation/further decompensation, (iii) acute-on-chronic liver failure (ACLF) and (iv) liver-related death. Univariate and multivariate multivariate competing risk regression models are shown. Liver transplantation and death were considered as competing risks for (i) and (iii), while liver transplantation and non-liver-related death were considered as competing risks for (ii) and (iv). Adjusted subdistribution hazard ratio (asHR) with 95% confidence interval (95%CI) and significance levels of multivariate Fine and Gray competing risk regression models for serum total cortisol: (i) asHR: 2.20 (95%CI 1.07-4.50), p=0.031; (ii) asHR: 2.23 (95%CI 1.27-3.91), p=0.005; (iii) asHR: 2.65 (95%CI 1.20-5.85), p=0.016; (iv) asHR: 2.25 (95%CI 0.89-5.71), p=0.087.

Parameter of interest	Univariate (unadjusted) analysis			Multivariate (adjusted) analysis		
**(i) bacterial infections**	**sHR**	**95%CI**	**p-value**	**asHR**	**95%CI**	**p-value**
**Serum free cortisol <4.8 ng/mL, yes/no**	1.82	0.89-3.71	0.099	2.20	1.07-4.50	**0.031**
**Age, 10 years**	1.16	0.88-1.53	0.300	-	-	-
**Sex (male)**	1.14	0.52-2.50	0.740	-	-	-
**Child Turcotte Pugh score, points**	1.09	0.93-1.28	0.310	-	-	-
**Creatinine, mg x dL^{-1}**	4.70	2.59-8.52	**<0.001**	4.54	2.31-8.95	**<0.001**
**Sodium, mmol x L^{-1}**	0.99	0.88-1.14	0.970	-	-	-
**HVPG, mmHg**	1.02	0.97-1.08	0.420	-	-	-
**C-reactive protein, mg x dL^{-1}**	1.29	1.02-1.64	**0.036**	1.23	0.96-1.57	0.096
**(ii) decompensation/further decompensation**						
**Serum free cortisol <4.8 ng/mL, yes/no**	2.00	1.08-3.70	**0.027**	2.23	1.27-3.91	**0.005**
**Age, 10 years**	0.88	0.69-1.13	0.330	-	-	-
**Sex (male)**	0.95	0.54-1.66	0.850	-	-	-

**Child Turcotte Pugh score, points**	1.30	1.16-1.47	**<0.001**	1.22	1.05-1.42	**0.008**
**Creatinine, mg x dL^{-1}**	2.88	1.72-4.81	**<0.001**	2.59	1.32-5.07	**0.005**
**Sodium, mmol x L^{-1}**	0.88	0.82-0.95	**<0.001**	0.91	0.85-0.98	**0.010**
**HVPG, mmHg**	1.13	1.08-1.18	**<0.001**	1.06	1.00-1.11	**0.046**
**C-reactive protein, mg x dL^{-1}**	1.60	1.31-1.97	**<0.001**	1.27	1.02-1.59	**0.035**
**(iii) ACLF**	**sHR**	**95%CI**	**p-value**	**asHR**	**95%CI**	**p-value**
**Serum free cortisol <4.8 ng/mL, yes/no**	1.88	0.94-3.79	0.076	2.65	1.20-5.85	**0.016**
**Age, 10 years**	1.09	0.83-1.44	0.540	-	-	-
**Sex (male)**	1.26	0.58-2.75	0.560	-	-	-
**Child Turcotte Pugh score, points**	1.26	1.09-1.47	**0.002**	1.37	1.13-1.64	**0.001**
**Creatinine, mg x dL^{-1}**	4.22	2.25-7.89	**<0.001**	3.63	1.76-7.45	**<0.001**
**Sodium, mmol x L^{-1}**	0.95	0.85-1.06	0.320	-	-	-
**HVPG, mmHg**	1.03	0.98-1.09	0.220	-	-	-
**C-reactive protein, mg x dL^{-1}**	1.23	0.95-1.58	0.110	-	-	-
**(iv) Liver-related death**						
**Serum free cortisol <4.8 ng/mL, yes/no**	1.57	0.71-3.48	0.270	2.25	0.89-5.71	0.087
**Age, 10 years**	1.10	0.78-1.55	0.590	-	-	-
**Sex (male)**	1.34	0.53-3.40	0.540	-	-	-
**Child Turcotte Pugh score, points**	1.28	1.09-1.51	**0.002**	1.29	1.04-1.60	**0.019**
**Creatinine, mg x dL^{-1}**	3.20	1.29-7.96	**0.012**	2.93	1.16-7.41	**0.023**
**Sodium, mmol x L^{-1}**	0.98	0.91-1.06	0.630	-	-	-
**HVPG, mmHg**	1.06	0.99-1.13	0.069	1.03	0.95-1.11	0.470
**C-reactive protein, mg x dL^{-1}**	1.27	0.92-1.76	0.140	-	-	-
